# Fibre Computer Enables More Accurate Recognition of Human Activity

**DOI:** 10.1007/s40820-025-01809-x

**Published:** 2025-06-06

**Authors:** Qianyi Cheng, Jianfeng Li, Qichong Zhang

**Affiliations:** 1 School of Materials Science and Engineering, Lanzhou Jiaotong University, Lanzhou, 730070 People’s Republic of China; 2 Key Laboratory of Multifunctional Nanomaterials and Smart Systems, Suzhou Institute of Nano-Tech and Nano-Bionics, Chinese Academy of Sciences, Suzhou, 215123 People’s Republic of China

**Keywords:** Single-fibre computer, Wearable electronics, Textile networks, Distributed inference

## Abstract

The advancement of fibre electronics is crucial for developing wearable smart textiles. However, traditional single-function fibres are typically limited to basic sensing and data collection capabilities, lacking effective computational and multimodal signal processing abilities, thus significantly restricting their potential in human activity recognition. Recently, Gupta et al*.* introduced an innovative single-fibre computer embedding eight microelectronic devices, integrating sensing, communication, and computation into a single fibre. Establishing a distributed cooperative fibre network substantially enhanced human activity recognition accuracy from 67% (single-fibre scenario) to 95%. This novel approach effectively addresses the limitations of conventional smart fibres, paving the way for multi-point sensing, edge-based inference, and real-time human–computer interactions in future intelligent textiles.

## Highlights

- Conventional microelectronics are scaled to the fibre level for information processing and storage in flexible, stretchable textiles.
- Compared to single-fibre systems, multi-fibre computational architectures with coordinating modules exhibit higher reliability and more comprehensive functionalities.

## Graphical abstract

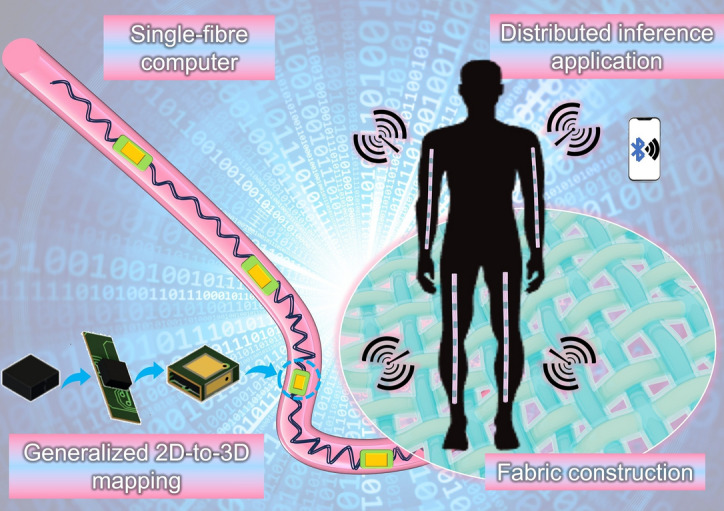

## Main Text

The development of fibre-based electronics is indispensable to the advancement of intelligent textiles, serving as a foundational bridge that connects conventional fabrics with next-generation wearable electronic systems [1, 2]. However, one of the most significant limitations of current smart textiles lies in the restricted functionality of individual fibre units, which typically lack embedded computational capabilities [3, 4]. This makes performing localized processing and real-time interpretation of complex biosignals difficult. Additionally, the spatial resolution of signal acquisition is often insufficient, limiting the system’s ability to accurately and comprehensively capture full-body motion dynamics. Moreover, communication between sensing nodes generally relies on rigid wiring, which reduces the textile’s mechanical flexibility and impedes efficient signal transmission, collaborative sensing, and distributed data fusion—all of which are essential for scalable, high-performance systems [5–7]. Compounding these challenges, most existing innovative textile platforms lack the architectural flexibility and scalability required to integrate and analyse multimodal inputs, such as mechanical, optical, and electrophysiological signals. Such limitations critically hinder the adaptability, intelligence, and practical deployment of next-generation smart textiles across diverse application scenarios.

To overcome these challenges, significant breakthroughs in fibre-level system integration are required. The latest study published in Nature by Gupta et al. reported an autonomous programmable elastic fibre computer [8]. It transformed the computing architecture of conventional chips to the fibre scale, and effectively integrating multiple electronic components within a single fibre—thereby imparting capabilities for sensing, computing, storage, and wireless communication—is a crucial strategy for addressing the current limitations of fibre electronics. This approach paves the way for a paradigm shift in wearable electronics and intelligent textiles. The single-fibre computer integrates eight microdevices via helical wiring during thermal drawing, consolidating full computing, sensing, storage, and communication capabilities within a single elastic fibre. The resulting functional fibre exhibits over 60% stretchability and is machine-washable, offering high elasticity alongside embedded computational capabilities.

**Fig. 1:**
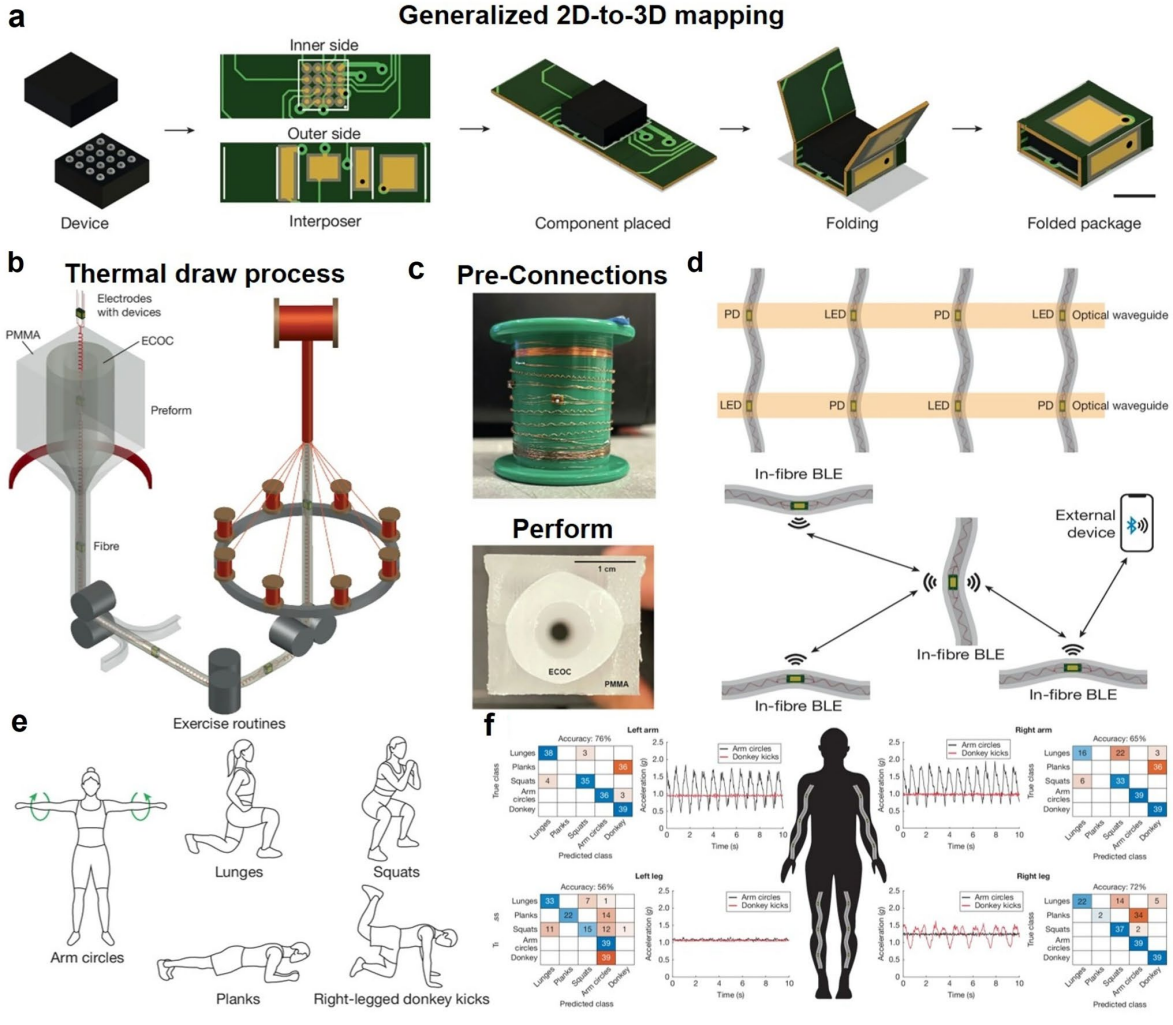
**a** Schematic of the flexible interposer method for device packaging. **b** Schematic of the thermal draw process used to fabricate the fibre computer with the subsequent braiding process. **c** Photograph of devices pre-connected on an I2C bus on a spool and preform with PMMA cladding encasing hollow ECOC cylinder. **d** Schematic of an optical and Bluetooth communication network of multiple fibre. **e** Schematics of the five exercise routines that aimed to record a training wearer. **f** Representative accelerometer magnitude signals for activity classification in wearable sensing systems

As illustrated in Fig. 1a, Gupta et al*.* introduced an ingenious foldable interposer strategy to translate the 2D electrical pad arrangements on individual microchips to 3D wire configurations within a fibre architecture. In this ingenious structural design, 2D microchips are encapsulated within a folded flexible interposer, where the inner pads are aligned with the chip pins and the outer pads are reconfigured into an axial array along the fibre surface. These outer pads serve as reliable connection points to fine copper microwires, enabling robust and scalable integration of microelectronic devices into the fibre architecture. Moreover, this 2D-to-3D mapping approach exhibits excellent versatility, accommodating microchips of varying sizes and functionalities, offering a universal solution for efficiently integrating chip-scale devices into fibre-based electronic systems.

To enhance the flexibility, elasticity, and mechanical durability of electronic fibre, Gupta et al. introduced innovative structural designs and material compositions in the fabrication of thermally drawn electronic fibre (Fig. 1b). In structural design, the strategy centred on changing the copper electrodes that connect the individual chips into a spiral spring configuration. Attributed to the helical structure with a diameter of 300 μm of the copper electrodes after plastic deformation, the electrodes can expand during stretching and recover during relaxation, ensuring high elasticity and flexibility of the electronic fibre (Fig. 1c). In material selection, the fibre drawing process starts with a double-layer preform of two distinct polymeric materials: an outer polymethyl methacrylate (PMMA) cladding for mechanical support and an inner hollow cylinder of thermoplastic cyclic olefin copolymer elastomer (ECOC) for elasticity, preventing uneven deformation during stretching (Fig. 1c). During fibre drawing, electronic components interconnected by helical copper microwires are encapsulated within an elastomeric cladding with a central bore (ECOC). Following thermal drawing, a coaxial single-fibre computer is produced. The helical architecture of the copper electrodes accommodates axial strain during and after fabrication, ensuring persistent stretchability, electrical integrity, and mechanical resilience. After removing the PMMA cladding, a soft ECOC fibre body remains, allowing the creation of a machine-washable elastic fibre that stretches over 60% without failure.

Due to the two built-in communication systems, the single-fibre computer can wirelessly transmit data to user terminals such as smartphones via a BLE unit, facilitating convenient information exchange (Fig. 1d). Optical communication between adjacent fibres is achieved through embedded LEDs and photodetectors, enabling rapid, low-latency data exchange over short distances. In parallel, BLE modules establish wireless links across extended ranges, facilitating flexible, scalable, and decentralized networking beyond direct line-of-sight constraints. This design overcomes the limitations of traditional fibre electronic devices that rely on rigid wiring, significantly enhancing mechanical reliability and durability. Moreover, it provides an efficient and robust technical solution for realizing textile-based networks and distributed intelligence, laying a critical foundation for the continued advancement of smart textiles. Each fibre computer consists of eight devices connected on a single I2C bus, including four sensors (a photodetector, a temperature sensor, a PPG sensor, and an accelerometer), a 32-bit microcontroller (MCU), two communication modules (an LED and a BLE unit), and the necessary power management devices. These devices work together to achieve data acquisition, data processing, storage, and result transmission functions. While the current design demonstrates effective fibre-to-fibre communication, challenges remain in optimizing communication speed, energy consumption, and bandwidth, particularly in multi-fibre cooperative networks. Improving the efficiency of inter-node information exchange will be critical for scaling up system performance. Future work could focus on developing higher-throughput, low-latency communication protocols tailored for textile-integrated networks to further enhance the scalability and intelligence of fibre-based systems.

To demonstrate the potential of the fabric network system for real-time monitoring of human activities, a single participant was instructed to perform a series of bodyweight exercises, such as squats, lunges, planks, arm circles, and right-leg donkey kicks (Fig. 1e). Researchers integrated four computationally capable smart fibre into a garment’s sleeves and pant legs, constructing a fabric-based distributed computing network via the embedded communication modules within each fibre (Fig. 1f). Each fibre computer runs an independently trained neural network. The system enables real-time recognition of human activities such as squatting, planking, arm rotation, and lunging through inter-node information sharing and collaborative inference. A standalone fibre computer achieves only 67% accuracy in recognizing human activities, whereas the textile-based architecture significantly boosts inference accuracy to 95%. This remarkable improvement underscores the immense potential of multi-fibre collaborative sensing and distributed reasoning, presenting a transformative approach for intelligent wearable systems that seamlessly integrate localized computation with networked decision-making to deliver robust, high-fidelity performance. However, the current demonstration primarily relies on a simple weighted voting strategy for distributed inference, and future efforts should explore more advanced distributed learning frameworks to further enhance the system’s autonomous adaptability and intelligent evolution. Approaches such as federated learning, decentralized training, or lightweight on-fibre model updating could offer promising pathways to unlock higher-level cognitive capabilities in fibre-based networks.

In conclusion, Gupta et al. proposed a single-fibre computer that enables textile-based networks and distributed inference. This novel single-fibre computer integrates multiple sensing elements within a single fibre, allowing for multimodal information acquisition at the fibre level. Thanks to its innovative fabrication strategy, the internal sensing network of the fibre can be dynamically reconfigured or updated according to application needs, endowing the system with programmability and upgradability. This work opens a promising pathway towards intelligent, reconfigurable, and highly integrated wearable textiles. The adoption of a foldable interposer approach effectively resolves the topological mismatch between two-dimensional microchips and the three-dimensional cylindrical geometry of fibre. This 2D-to-3D mapping strategy exhibits high versatility, accommodating various microelectronic devices with varying package sizes and pin configurations. Furthermore, the optical and radio-frequency communication strategy employed in the single-fibre computer enables wireless data transmission within textile architectures, eliminating the need for embedded wiring. This approach not only enhances the mechanical flexibility and wearability of the fabric but also facilitates modular integration, low-latency communication, and scalable network formation, thereby advancing the development of truly wireless, intelligent textile systems. This study offers an innovative approach and technological paradigm for advancing smart textiles towards system-level integration and edge computing, paving the way for the next generation of wearable technologies to enter a truly intelligent and interactive era. Building on this foundation, future research directions become increasingly important. We look forward to continued research efforts that will focus on further enhancing the ability of multi-fibre cooperative and performance of functional fibre, thereby broadening their applicability across a wider range of scenarios. Such advancements are expected to drive the development of next-generation single-fibre computer, opening new possibilities for human-centred information interaction.
